# Raman liquid biopsy: a new approach to the multiple sclerosis diagnostics

**DOI:** 10.3389/fneur.2025.1516712

**Published:** 2025-04-16

**Authors:** Anna Neupokoeva, Ivan Bratchenko, Lyudmila Bratchenko, Elena Khivintseva, Igor Shirolapov, Natalia Shusharina, Matvei Khoimov, Valery Zakharov, Alexander Zakharov

**Affiliations:** ^1^Department of Medical Physics, Mathematics and Computer Science, Samara State Medical University, Samara, Russia; ^2^Laser and Biotechnical Systems Department, Samara National Research University, Samara, Russia; ^3^Department of Neurology and Neurosurgery, Samara State Medical University, Samara, Russia; ^4^Department of Physiology, Samara State Medical University, Samara, Russia; ^5^Research Institute of Neurosciences, Samara State Medical University, Samara, Russia; ^6^Baltic Center for Neurotecnology and Artificial Intelligence, Immanuel Kant Baltic Federal University, Kaliningrad, Russia

**Keywords:** multiple sclerosis, surface enhanced Raman spectroscopy, projection on latent structures-discriminant analysis, blood serum, diagnostic test

## Abstract

**Background/objectives:**

Despite the prevalence of multiple sclerosis, there is currently no biomarker by which this disease can be reliably identified. Existing diagnostic methods are either expensive or have low specificity. Therefore, the search for a diagnostic method with high specificity and sensitivity, and at the same time not requiring complex sample processing or expensive equipment, is urgent.

**Methods:**

The article discusses the use of blood serum surface enhanced Raman spectroscopy in combination with machine learning analysis to separate persons with multiple sclerosis and healthy individuals. As a machine learning method for Raman spectra processing the projection on latent structures-discriminant analysis was used.

**Results:**

Using the above methods, we have obtained possibility to separate persons with multiple sclerosis and healthy ones with an average specificity of 0.96 and an average sensitivity of 0.89. The main Raman bands for discrimination against multiple sclerosis and healthy individuals are 632, 721–735, 1,048–1,076 cm^−1^. In general, the study of the spectral properties of blood serum using surface enhanced Raman spectroscopy is a promising method for diagnosing multiple sclerosis, however, further detailed studies in this area are required.

## 1 Introduction

Multiple sclerosis (MS) is a chronic, autoimmune disease of the central nervous system (CNS) that affects millions of people worldwide. The relevance of studying MS is explained by its high prevalence and significant consequences for the patient life quality. According to the World Health Organization, more than 2.8 million cases of the disease were recorded in 2023. At the same time, the incidence continues to grow, especially in regions with temperate and cold climates, such as Northern Europe, North America and Australia (1, 2). MS is one of the most common causes of non-traumatic disability among young people (aged 18–40 years) (2, 3), which creates additional social and economic challenges for health systems around the world (4–6).

The MS course is diverse but the most common forms are recurrent and progressive. Under a long disease continuance the recurrent course may turn into a progressive one (7, 8). With a disease duration of more than 5 years in 25% of patients, MS passes into secondary progressive MS, after 10 years this form occurs in 50% of patients and after 20 years covers more than 70% (9–11). At the beginning of the disease, the so-called clinically isolated or radiologically isolated syndrome may occur, i.e., conditions accompanied by only one clinical attack or single focal lesions of the central nervous system without clinical manifestations (12–14). In 10% of patients, the disease gradually progresses from the very beginning and is regarded as primary progressive multiple sclerosis (15, 16). The reason for such a variety of the disease course is varying intensity of the demyelination and neurodegeneration processes. The existing disease therapy is aimed at stabilizing the disease progression or preventing exacerbations (17, 18).

The MS diagnosis is based on a combination of clinical, instrumental and laboratory data, since there is no specific test for this disease. Magnetic resonance imaging (MRI) is the main imaging method in the MS diagnosis which allows to identify demyelination lesions in the brain and spinal cord. The use of a contrast agent (most often gadolinium) helps to detect active inflammatory processes and new lesions. MRI allows to diagnose the presence of subclinical lesions that do not manifest themselves symptomatically but are important for confirming the diagnosis (19). MRI is the most reliable method of diagnosis and assessment of the course of the disease because it allows you to identify subclinical focal lesions of the central nervous system. However, despite the high availability, MRI examinations are not performed more than twice a year.

Other instrumental methods such as evoked potentials, optical coherence tomography are also actively used (20, 21). These methods are more accessible because they are less financially costly. However, their disadvantage should be considered low specificity, which does not allow them to be used as a routine method for evaluating the effectiveness of MS therapy.

Among the promising methods of instrumental diagnostics quantum sensitive MRI (qMRI) should be noted. This method allows microstructural changes measuring in brain tissues by microscopic parameters analysis such as axon density, changes in white matter and myelin content, that helps to clarify the disease stage and the treatment effectiveness (22) diagnostic methods are also being developed, which in some cases complement instrumental methods or serve as an independent diagnostic tool. For example, oligoclonal bands are present in 85–95% of MS patients and are an important marker for confirming the diagnosis (23). Although specific biomarkers for the diagnosis of MS in peripheral blood do not exist now, the light neurofilament chain and glutamate may reflect the degree of neuronal damage and disease progression. It is expected that these markers can become useful tools for early diagnosis and monitoring of the disease (24).

Thus, the search for an affordable diagnostic method with high sensitivity and specificity is carried out. The method should be based on the analysis of biological data that does not require prior preparation or the use of extremely expensive equipment.

Optical methods are well suited for solving this problem. In particular Raman spectroscopy allows detecting the individual metabolites content in the studied sample and also has sensitivity to the structural features of the researched substances. Recently, the effectiveness of Raman spectra using for the detection of various diseases has been shown (25, 26), and the differentiation between a healthy state and pathology occurs on the basis of mathematical processing of the spectrum as a whole, and not according to the specific biomarker content (27).

Attempts to use spectral analysis methods to diagnose MS have been presented for blood analysis (28, 29) using ATR-FTIR (Attenuated Total Reflectance Fourier Transform Infrared) spectroscopy, as well as for the analysis of retinal tissues using Raman spectroscopy (30). These studies showed the prospects of spectroscopic techniques using for the MS diagnosis by analyzing the quantitative content of carotenoids in the analyzed samples. At the same time, the authors did not make reference measurements of carotenoids using standard chemical methods, so the possibility of diagnosing MS through spectral analysis of carotenoids is still a hypothesis that requires further verification. Some MS research, metabolomic profiling has revealed alterations in glycerophospholipid and linoleic acid pathways, with potential for diagnosis and disease monitoring (31). Cerebrospinal fluid analysis in MS patients has identified phospholipid alterations correlating with clinical data, as well as elevated glutamate levels (32). However, a recent review highlights the key role of ergothioneine in label-free SERS spectra of biofluids, suggesting that many past studies may have inadvertently attributed its spectral features to other molecules. This revelation emphasizes the need for re-evaluation of previous SERS-based biofluid analyses in various clinical conditions (33).

In order to study in more detail, the metabolism changes of patients with MS more complex spectral approaches should be used, for example, such as SERS (surface-enhanced Raman spectroscopy). In the SERS study, nanostructures of noble metals are used to amplify the signal, which leads to an increase in the recorded Raman scattering signal by thousands of times (34). Therefore, the purpose of this work is to investigate the possibility of SERS using for the human blood serum analysis in order to diagnose multiple sclerosis. The PLS-DA (projection on latent structures-discriminant analysis) method was used to analyze the recorded spectral dependencies and classify the analyzed samples.

## 2 Materials and methods

The study was approved by the Ethics Committee of the Federal State Budgetary Educational Institution of Higher Education Samara State Medical University of the Ministry of Health of the Russian Federation, Protocol No 52 from 12 December 2023.

The study included 48 patients with an active and stable course of MS, established in accordance with the McDonald criterion of 2017 (35), as well as 30 healthy volunteers without any chronic diseases. The disease activity was established based on the presence of one exacerbation during the previous year or two exacerbations during the last 2 years in patients with relapsing–remitting and secondary-progressive with exacerbations of MS. In patients with a primary progressive course, activity was determined by the disease progression during the last year. Patients with a stable disease course did not have exacerbations or an increase in the disease during the specified period of time (35). In patients with an active course, increasing neurological deficit was observed by at least 2 points on one of the functional scales (for example, visual, stem, pyramidal, sensory, coordination) or at least 1 point on two functional systems. There could also be a progression of disability on the EDSS scale (36): an increase of at least 1 point if the initial EDSS was below 4.0 points or an increase of at least 0.5 points if the initial EDSS was 4.0 or higher (37). In patients with a progressive course, there is an increase in neurological deficit by 1 point on the EDSS scale (38). Neurological examination and assessment according to the EDSS scale (36) was done by a certified neurologist with experience in managing MS patients for more than 10 years. Venous blood sampling in a volume of 9 mL was performed in patients with compliance with the rules of asepsis and antiseptics.

Description of the group of patients with MS is presented in Table 1. The EDSS score is given in the form of a minimum-maximum range, the average value is given in parentheses.

**Table 1:** Clinical characteristics of patients with MS.

EDSS functional systems							
Vision mean (min–max)	Brainstem mean (min–max)	Pyramidal mean (min–max)	Cerebellar mean (min–max)	Sensory mean (min–max)	Bowel and bladder mean (min–max)	Cerebral functions mean (min–max)	Score mean (min–max)
0 (0–2)	1 (0–3)	2 (1–4)	2 (0–4)	1 (0–3)	1 (0–3)	1 (0–2)	3.0 (1.0–7.0)

In 18 patients, activity was observed during MS; in 30 patients, activity was not observed over the past 2 years. One patient had a secondary progressive course with exacerbations of MS, the remaining 47 patients had a relapsing–remitting course of the disease.

As a method of diagnosing MS, we proposed the surface-enhanced Raman spectra registration from blood serum and further spectra processing. The main group (“Target”) included 48 patients with an established diagnosis of MS. The control group (“Control”) included persons of appropriate age without signs of neurodegenerative diseases (healthy donors from a blood transfusion station). The distribution of subjects by gender and age is shown in Table 2.

**Table 2:** Characteristics of the analyzed samples.

Group	Gender (M/F)	Age [min-max(mean)]	Number of spectra
Target	48 (23/25)	20–53 (32)	144
Control	30 (14/16)	29–50 (35)	88

SERS spectra registration was performed based on the approach described in our previous publications (39). Briefly, silver structures based on dried silver colloid are utilized to achieve surface enhancement of Raman scattering in the near infrared range. A silver colloid was obtained by reduction from an aqueous solution of silver nitrate with sodium citrate at the temperature of 95°C for 20 min. The resulting colloidal solution was poured onto an aluminum foil and dried at room temperature until completely dry.

For SERS analysis, each serum sample was dropped in a volume of 1.5 μL on aluminum foil with a layer of silver structures and dried for 30 min. The analysis of the serum spectral characteristics was carried out using an experimental stand consisting of a spectrometric system (EnSpectr R785, Spektr-M, Chernogolovka, Russia) and a microscope (ADF U300, ADF, China). The spectra were excited in the near infrared range using a laser module with the center wavelength of 785 nm. Human serum was analyzed at the laser power of 10 mW. The spectra were recorded with the exposure time of 4 s x4 times. In the current work, we used an experimental setup consisting of a spectrometric system (EnSpectr R785, Spektr-M, Chernogolovka, Russia) based on a CCD detector and a microscope (ADF U300, ADF, China). The setup does not contain any additional gratings, slits, or other optical elements. The distance between the sample surface and the objective was adjusted manually using the focusing screw of the microscope to achieve a distance equal to the focal length of the objective. The recorded raw spectrum for each sample is an automatic sequential recording of four spectra with subsequent averaging to compensate for shot noise. Before recording the spectral characteristics of the serum sample under study, a preliminary recording of the surrounding background signal was made. After that, the background component was automatically subtracted from the subsequent recorded serum spectra using the algorithm built into the EnSpectr program. The background component refers to the background in the room and the noise of the optical system recorded by the spectrometric system used in the absence of laser radiation. The spectral contribution of the substrate was also recorded to control for possible spectral contribution to the spectra of serum samples.

Immediately before the registration of the tested serum sample spectral characteristics, a preliminary recording of the environing background signal was performed. After that, the background component was automatically subtracted from the subsequent recorded serum spectra using the algorithm built in the EnSpectr software. 3 spectra were registered for each sample.

Preprocessing of surface-enhanced Raman spectra of serum consisted of several successive steps: noise smoothing, removal of autofluorescent background and normalization. Smoothing of raw spectra was performed using the Savitzky–Golay filter with a filter window width of 15, the first order of the polynomial used for smoothing and a zero-order derivative (no derivative). Then, the smoothed spectra were subjected to removal of autofluorescent background by baseline correction (15th polynomial degree). Spectral characteristics of serum were normalized using the Standard Normal Variate (SNV) algorithm. The SNV algorithm first implements the centering of each spectrum and then scaling by dividing by the standard deviation.

Discrimination of neurological patients group (“Target”) vs. healthy donors (“Control”) by spectral characteristics of serum SERS was based on PLS-DA implementation (40). The analyzed data set is divided randomly: 80% of the subjects of each group for model training (training sample) and 20% of the subjects of each group for model testing (test sample). To select the optimal parameters for building the model on the training sample, k-fold cross-validation (k = 7) was performed. Based on the selected parameters, the model was trained on a training sample, then applied to classify the test sample. Then the division into training and test samples was repeated 29 more times (a total of 30 iterations).

When constructing the models, the importance of predictors in accomplishing the classification task was assessed by means of the distribution of variable importance in the constructed model. The variable importance distribution analysis makes it possible to define which spectral bands and associated serum components are characterized by the differences in the accomplished classification task. Moreover, the estimation of the variable importance distribution can circumvent the case when the model is a “black box” and is erroneously associated with noise. The variables importance distributions for PLS and PLS-DA models were calculated by a standard algorithm as a weighted sum of the squared correlations between the PLS-DA components and the variable (40).

## 3 Results

**Figure 1:**
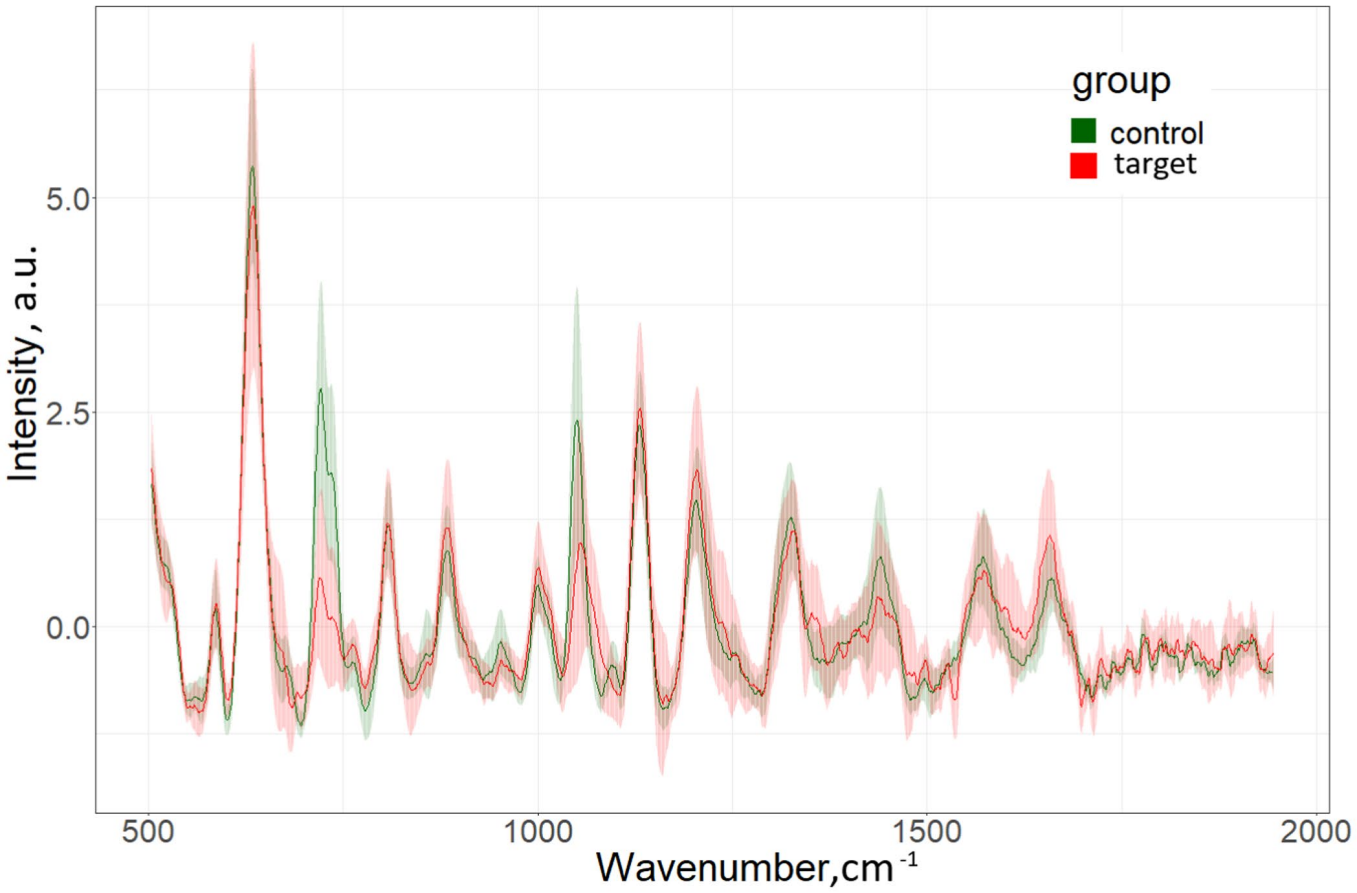
Mean SERS spectra and standard deviation for the target and control groups.

The characteristic differences in the averaged spectra for the target group (patients with MS) and for the control group (persons without MS) are shown in Figure 1.

**Figure 2:**
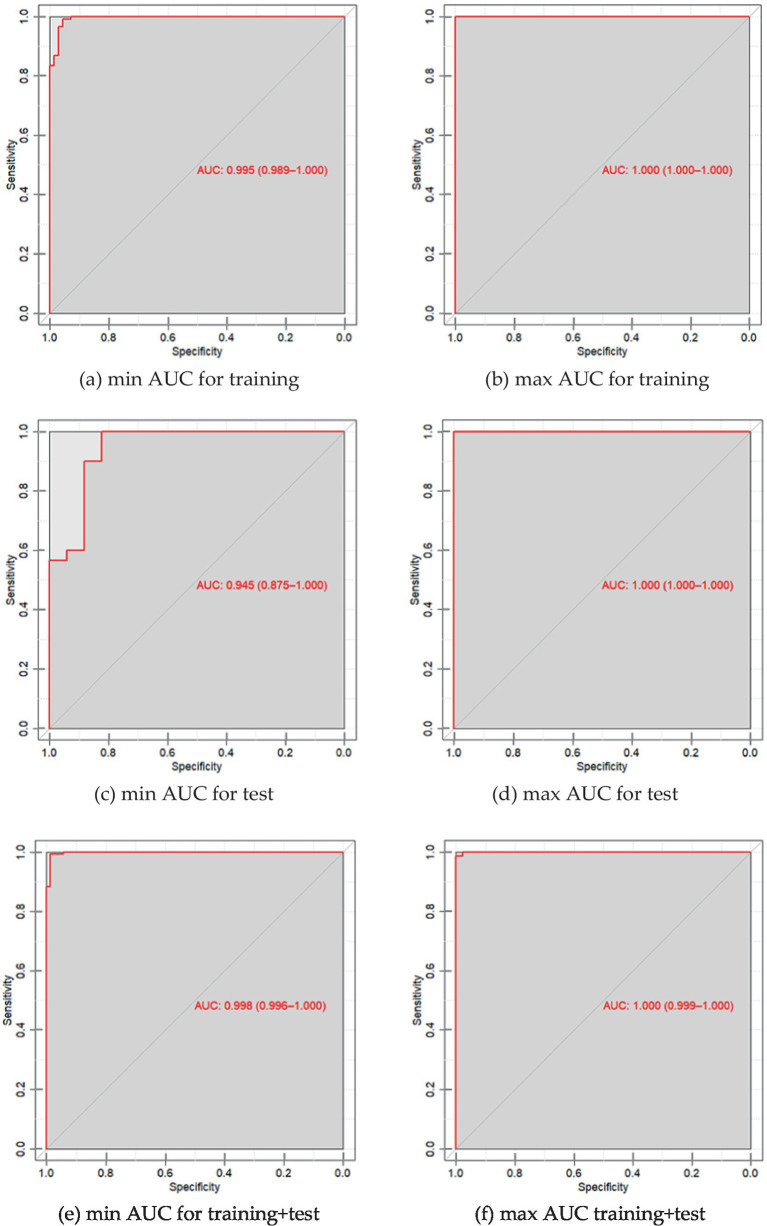
**(a)** ROC curve of PLS-DA discrimination of serum spectral characteristics of patients with neurological diseases and the control group with a minimum AUC value for 30 iterations of model construction for training samples; **(b)** ROC curve of PLS-DA discrimination of serum spectral characteristics of patients with neurological diseases and the control group with a maximum AUC value of 25 iterations of building models for training samples; **(c)** ROC is the PLS-DA curve of discrimination of serum spectral characteristics of patients with neurological diseases and the control group with a minimum AUC value for 30 iterations of model construction for test samples; **(d)** ROC is the PLS-DA curve of discrimination of serum spectral characteristics of patients with neurological diseases and the control group with a maximum AUC value for 25 iterations of model construction for test samples; **(e)** ROC is the PLS-DA curve of discrimination of serum spectral characteristics of patients with neurological diseases and the control group with a minimum AUC value for 30 iterations of constructing models for the entire dataset; **(f)** ROC is the PLS-DA curve of discrimination of serum spectral characteristics of patients with neurological diseases and the control group with a maximum AUC value for 25 iterations of constructing models for the entire dataset.

Data on the specificity, sensitivity and accuracy of the proposed discrimination method are presented in Table 3; Figure 2.

**Table 3:** Characteristics of PLS-DA discrimination neurological patients (“Target”) vs. healthy donors (“Control”) according to the serum spectral characteristics.

Subsample	Specificity mean (min–max)	Sensitivity mean (min–max)	Accuracy mean (min–max)	ROC AUC mean (min–max)
Training	0.99 (0.96–1.0)	0.97 (0.96–0.99)	0.98 (0.96–0.99)	0.99 (0.99–1.00)
Test	0.96 (0.77–1.0)	0.89 (0.72–1.0)	0.94 (0.81–1.0)	0.98 (0.95–1.00)

**Figure 3:**
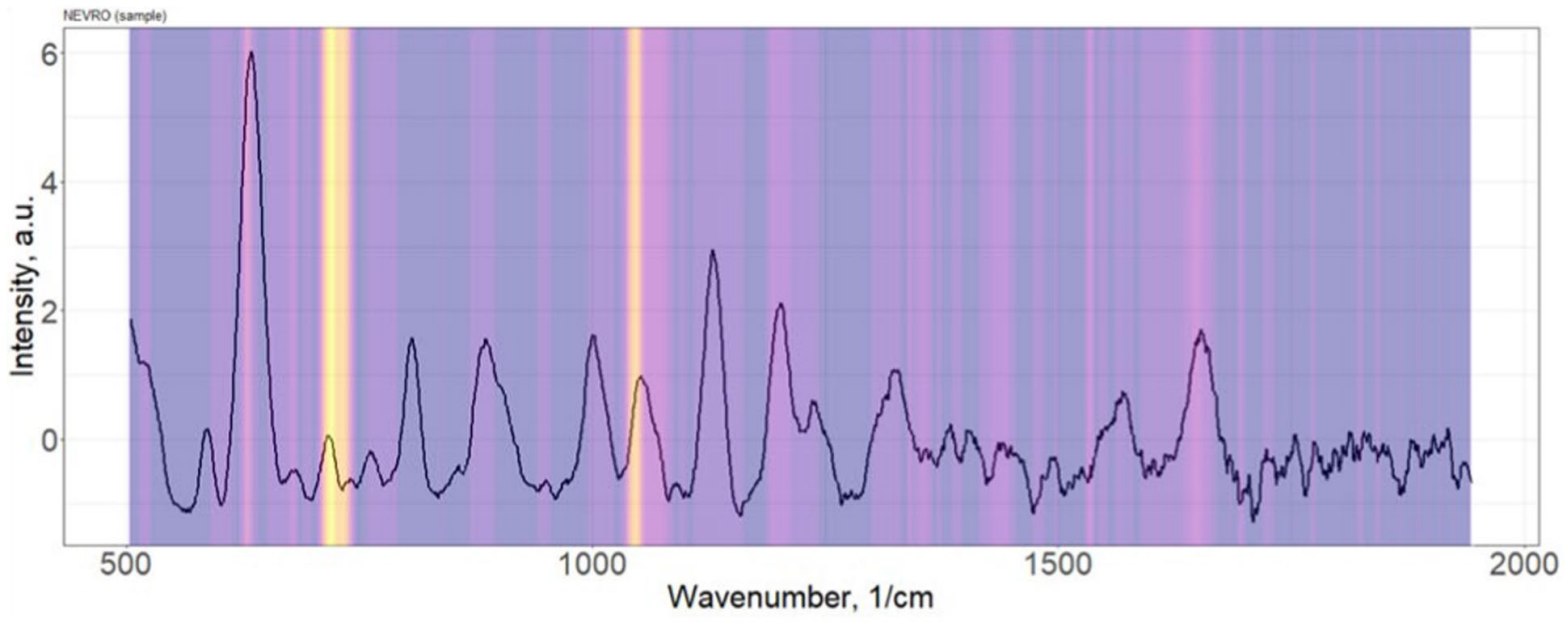
Distribution of the importance of variable serum spectral characteristics in the construction of the PLS-DA discrimination model neurological patients (“Target”) vs. healthy donors (“Control”).

Distribution of the importance of variable serum spectral characteristics in the construction of the PLS-DA discrimination model neurological patients (“Target”) vs. healthy donors (“Control”) is shown in Figure 3.

According to methodology proposed in our previous studies the SNR for the presented spectra was not less than 50 (40).

The most significant Raman bands of the serum spectrum for the construction of discrimination “Target” vs. “Control” (position of maxima): 632, 721–735, 1,048–1,076 cm^−1^. Single band intensity analysis demonstrates only about 60–65% accuracy of discrimination. As example, 632 cm^−1^ band provides 63% accuracy.

**Figure 4:**
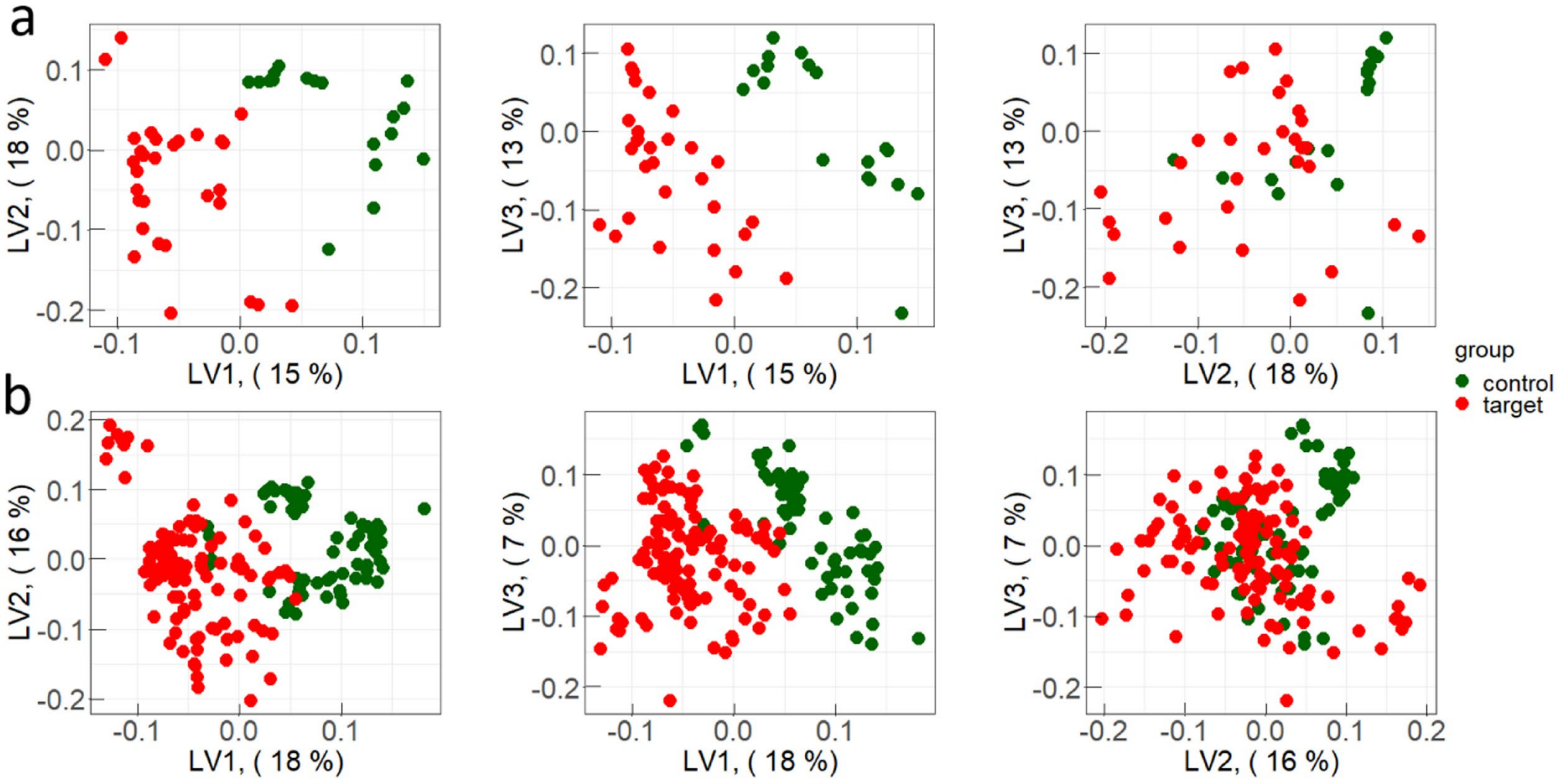
Examples of PLS-components scores (X-scores) plot for one iteration of constructing discrimination models for: **(a)** test sample and **(b)** train sample.

Representative examples of PLS-components scores (X-scores) plot for one iteration of constructing discrimination models of spectral data are presented in Figure 4.

## 4 Discussion

In comparison with works suggesting the ATR-FTIR using (28, 29) for the diagnosis of MS, in this work almost 3 times more patients with MS were studied, which provides more reliable data on the possibility of spectral blood serum features using for the diagnosis of MS.

The obtained accuracy is slightly higher than the accuracy achieved by ATR-FTIR using and in any case, all conclusions about the possibilities of MS spectral diagnosis should be tested on large cohorts of patients.

Interestingly, the authors of the works (28, 29) conclude that the observed differences between plasma samples of patients with MS and healthy people lie in the areas of 1,005 and 1,150 cm^−1^, and are associated with changes in the composition of carotenoids. In our study, the detection of MS patients occurs due to the Raman bands 632, 721–735 and 1,048–1,076 cm^−1^. Body tissues research during the MS development show various spectral bands that can be used for diagnostic purposes. For example, in the work (41) using coherent anti-Stokes Raman scattering, the authors showed changes in the concentration of myelin lipids in tissues during the MS development. In other paper (30), the authors used conventional Raman spectroscopy to analyze MS patient retinal tissues and recorded changes in the antioxidants concentration in the studied tissues.

The Raman spectral ranges of 623, 721–735, and 1,048–1,076 cm^−1^ can be associated with metabolites relevant to the diagnosis of multiple sclerosis (MS). Specifically, 623 nm corresponds to C-H bond vibrations, which can indicate changes in lipids such as phospholipids and cholesterol, components of the myelin sheath (42, 43). Alterations in these lipids can serve as markers of myelin damage during demyelination, a hallmark of MS.

The 721–735 cm^−1^ range is linked to amide bond vibrations, reflecting changes in proteins and peptides, such as myelin basic proteins (MBP), which degrade during inflammation (44).

Finally, 1,048–1,076 cm^−1^ includes vibrations of carbohydrates and lipids, which may signal changes in cell membranes and glycoproteins involved in inflammation and immune cell activation in MS. These spectral features can be used for monitoring disease progression and assessing inflammatory processes (45).

Currently, there is a tendency to use not just one marker for early diagnosis of diseases, but information on the biological liquids indicators in general (46, 47). At the same time, Raman spectroscopy in combination with machine learning shows the prospects for developing an accurate, inexpensive, fast and non-invasive method of universal medical diagnostics (48). However, still some challenges must be eliminated prior to translation of Raman spectroscopy into clinical applications (49).

It has been shown that under MS blood viscosity changes (50) and a perfusion modification in the demyelination zones is observed (51). The blood rheological properties changes lead to changes in the plasma protein composition. Therefore, Raman spectroscopy as a method for MS diagnosing is promising since the spectra are sensitive to both quantitative and qualitative changes in the components of bioliquids. On the other hand, in many fields of medicine, there is a tendency to replace a certain part of expensive, time-consuming or invasive diagnostic methods with faster and cheaper tools based on the Raman spectra analysis.

Some works (52) demonstrated the possibilities of neural networks application to classify blood serum Raman spectra for the identification of the cardiovascular system pathologies. The same approach may be applied to the diagnosis of neurological dis-eases. Xiong et al. (53) demonstrated 88% accuracy for the classification of healthy controls, mild cognitive impairment, Alzheimer’s disease, and Non-Alzheimer’s dementia; an accuracy of 90% for the classification of healthy controls, elderly depression, and elderly anxiety.

Our results show that the serum Raman spectra reflect changes in blood composition of MS patients compared with healthy individuals. The use of PLS-DA as a discrimination method ensures high specificity, sensitivity and accuracy, and also avoids overfitting classification models. This allows us to propose Raman spectroscopy as a method of MS prediagnosis before sending the patient to MRI. In the future, it is planned to study the question about effectiveness of Raman spectroscopy in combination with deep learning methods for the MS dynamics analysis. A positive answer to this question would allow to use this method for the treatment effectiveness evaluation between protocol MRIs, which are provided twice a year.

## 5 Conclusion

This study investigated the application of surface-enhanced Raman spectroscopy of serum combined with machine learning-based analysis to differentiate multiple sclerosis patients from healthy individuals (projection on latent structures-discriminant analysis was used to process the Raman spectra). The results demonstrate the high potential of the proposed method for discrimination between individuals with multiple sclerosis and healthy people, which is confirmed by the obtained data on average specificity (0.96) and average sensitivity (0.89). The accuracy of the proposed method was achieved at the level of 94%, which is higher than previous studies by some other scientists. The main Raman bands allowing to differentiate multiple sclerosis patients from healthy people were obtained at 632, 721–735 and 1,048–1,076 cm^−1^.

It is noted that the application of SERS in the diagnosis of MS offers promising advancements in the field of medical diagnostics. The method shows high potential due to its ability to differentiate between healthy individuals and MS patients with significant accuracy, sensitivity, and specificity. This study demonstrated that SERS can be effectively combined with machine learning algorithms, particularly the PLS-DA, to achieve robust classification of blood serum samples. Compared to traditional diagnostic methods like MRI or evoked potentials, Raman spectroscopy presents an affordable, fast, and minimally invasive alternative that could potentially bridge the gaps in current diagnostic protocols. Specifically, it could be used as a preliminary screening tool or in conjunction with other methods to monitor disease progression and therapeutic effectiveness between more invasive MRI assessments. The findings underline the importance of further studies to enhance the applicability of this technique in clinical practice, including expanding patient cohorts and identifying key molecular markers influencing the Raman spectra. With further refinement, SERS could serve as an essential tool for the early diagnosis of MS, aiding in timely intervention and better patient outcomes.

In general, it can be said that the study of the blood serum spectral properties using SERS is a promising method for MS diagnosing, however, further more detailed studies in this area are required. It is necessary to increase the cohort of analyzed patients, as well as try to establish which compounds have the greatest influence on the shape of the recorded spectrum.

## Data Availability

The raw data supporting the conclusions of this article will be made available by the authors, without undue reservation.

## References

[1] Thompson AJ Baranzini SE Geurts J Hemmer B Ciccarelli O. Multiple sclerosis. Lancet. (2018) 391:1622–36. doi: 10.1016/S0140-6736(18)30481-1, PMID: 29576504

[2] Walton C King R Rechtman L Kaye W Leray E Marrie RA. Rising prevalence of multiple sclerosis worldwide: insights from the atlas of MS, third edition. Mult Scler. (2020) 26:1816–21. doi: 10.1177/1352458520970841, PMID: 33174475 PMC7720355

[3] Koch-Henriksen N Sørensen PS. The changing demographic pattern of multiple sclerosis epidemiology. Lancet Neurol. (2010) 9:520–32. doi: 10.1016/S1474-4422(10)70064-8, PMID: 20398859

[4] Browne P Chandraratna D Angood C Tremlett H Baker C Taylor BV. Atlas of multiple sclerosis 2013: a growing global problem with widespread inequity. Neurology. (2014) 83:1022–4. doi: 10.1212/WNL.0000000000000768, PMID: 25200713 PMC4162299

[5] Zakharov AV Afrosina EY Khivintseva EV Antipov OI. The quality of nighttime sleep in patients with multiple sclerosis. Z Nevrol Psikhiatr Im SS Korsakova. (2016) 116:41–3. doi: 10.17116/jnevro20161162241-43, PMID: 27070360

[6] Zakharov AV Vlasov IV Poverennova IE Khivintseva EV Antipov OI. Posture disorders in patients with multiple sclerosis. Zh Nevrol Psikhiatr Im S S Korsakova. (2014) 114:55–8. PMID: 24662358

[7] Péran P Nemmi F Dutilleul C Finamore L Falletta Caravasso C Troisi E. Neuroplasticity and brain reorganization associated with positive outcomes of multidisciplinary rehabilitation in progressive multiple sclerosis: a fMRI study. Mult Scler Relat Disord. (2020) 42:102127. doi: 10.1016/j.msard.2020.102127, PMID: 32438326

[8] Tavazzi E Cazzoli M Pirastru A Blasi V Rovaris M Bergsland N. Neuroplasticity and motor rehabilitation in multiple sclerosis: a systematic review on MRI markers of functional and structural changes. Front Neurosci. (2021) 15:707675. doi: 10.3389/fnins.2021.707675, PMID: 34690670 PMC8526725

[9] Allington D. New approaches in the management of multiple sclerosis. Drug Des Devel Ther. (2010) 343:9331. doi: 10.2147/DDDT.S9331 PMC2998807 21151622

[10] Inojosa H Proschmann U Akgün K Ziemssen T. A focus on secondary progressive multiple sclerosis (SPMS): challenges in diagnosis and definition. J Neurol. (2021) 268:1210–21. doi: 10.1007/s00415-019-09489-5, PMID: 31363847

[11] Scalfari A Neuhaus A Daumer M DeLuca GC Muraro PA Ebers GC. Early relapses, onset of progression, and late outcome in multiple sclerosis. JAMA Neurol. (2013) 70:214–22. doi: 10.1001/jamaneurol.2013.599, PMID: 23407713

[12] Khabirov FA Vlasov IV Esin RG Kochergina OS Babicheva NN Zakharov AV. Influence of complex rehabilitation on the social adaptability and quality of life in people with multiple sclerosis. Zh Nevrol Psikhiatr Im S S Korsakova. (2009) 109:138–41. PMID: 19891359

[13] Thrower BW. Clinically isolated syndromes: predicting and delaying multiple sclerosis. Neurology. (2007) 68:85. doi: 10.1212/01.wnl.0000277704.56189.85 17562845

[14] Zakharov AV Khinivtseva EV Poverennova IE Gindullina EA Vlasov IV Sineok EV. Assessment of the risk of the transition of a monofocal clinically isolated syndrome to clinically definite multiple sclerosis. Zh Nevrol Psikhiatr Im S S Korsakova. (2013) 113:28–31. PMID: 23528591

[15] Lezhnyova V Davidyuk Y Mullakhmetova A Markelova M Zakharov A Khaiboullina S. Analysis of herpesvirus infection and genome single nucleotide polymorphism risk factors in multiple sclerosis, Volga federal district, Russia. Front Immunol. (2022) 13:1010605. doi: 10.3389/fimmu.2022.1010605, PMID: 36451826 PMC9703080

[16] Rice CM Cottrell D Wilkins A Scolding NJ. Primary progressive multiple sclerosis: progress and challenges. J Neurol Neurosurg Psychiatry. (2013) 84:1100–6. doi: 10.1136/jnnp-2012-304140, PMID: 23418213

[17] Boyko AN Khachanova NV Melnikov MV Sivertseva SA Evdoshenko EP Spirin NN. New directions of immunocorrection in multiple sclerosis. Z. Nevrol. Psikhiatr. Im. S.S. Korsakova. (2020) 120:103. doi: 10.17116/jnevro2020120021103, PMID: 32307419

[18] Poverennova IE Vlasov IV Zakharov AV Kuznetsova NI Romanova TV Katsnel’son VM. Some problems in rehabilitating disabled people with multiple sclerosis in agencies for social protection. Zh Nevrol Psikhiatr Im S S Korsakova. (2009) 109:129–34. PMID: 19891357

[19] Charil A Yousry TA Rovaris M Barkhof F De Stefano N Fazekas F. MRI and the diagnosis of multiple sclerosis: expanding the concept of “no better explanation.”. Lancet Neurol. (2006) 5:841–52. doi: 10.1016/S1474-4422(06)70572-5 16987731

[20] Klistorner A Graham EC Yiannikas C Barnett M Parratt J Garrick R. Progression of retinal ganglion cell loss in multiple sclerosis is associated with new lesions in the optic radiations. Eur J Neurol. (2017) 24:1392–8. doi: 10.1111/ene.13404, PMID: 28799222

[21] Petzold A Balcer LJ Calabresi PA Costello F Frohman TC Frohman EM. Retinal layer segmentation in multiple sclerosis: a systematic review and meta-analysis. Lancet Neurol. (2017) 16:797–812. doi: 10.1016/S1474-4422(17)30278-8, PMID: 28920886

[22] Fujita S Yokoyama K Hagiwara A Kato S Andica C Kamagata K. 3D quantitative synthetic MRI in the evaluation of multiple sclerosis lesions. AJNR Am J Neuroradiol. (2021) 42:471–8. doi: 10.3174/ajnr.A6930, PMID: 33414234 PMC7959431

[23] Deisenhammer F Zetterberg H Fitzner B Zettl UK. The cerebrospinal fluid in multiple sclerosis. Front Immunol. (2019) 10:726. doi: 10.3389/fimmu.2019.00726, PMID: 31031747 PMC6473053

[24] Novakova L Zetterberg H Sundström P Axelsson M Khademi M Gunnarsson M. Monitoring disease activity in multiple sclerosis using serum neurofilament light protein. Neurology. (2017) 89:2230–7. doi: 10.1212/WNL.0000000000004683, PMID: 29079686 PMC5705244

[25] Kong K Kendall C Stone N Notingher I. Raman spectroscopy for medical diagnostics — from in-vitro biofluid assays to in-vivo cancer detection. Adv Drug Deliv Rev. (2015) 89:121–34. doi: 10.1016/j.addr.2015.03.009, PMID: 25809988

[26] Li B Ding H Wang Z Liu Z Cai X Yang H. Research on the difference between patients with coronary heart disease and healthy controls by surface enhanced Raman spectroscopy. Spectrochim Acta A Mol Biomol Spectrosc. (2022) 272:120997. doi: 10.1016/j.saa.2022.120997, PMID: 35149484

[27] Tuchin VV. Multimodal optical diagnostics of Cancer. Cham: Springer International Publishing AG (2020).

[28] Chrabąszcz K Kołodziej M Roman M Pięta E Piergies N Rudnicka-Czerwiec J. Carotenoids contribution in rapid diagnosis of multiple sclerosis by Raman spectroscopy. Biochim Biophys Acta Gen Subj. (2023) 1867:130395. doi: 10.1016/j.bbagen.2023.130395, PMID: 37271406

[29] Kołodziej M Chrabąszcz K Pięta E Piergies N Rudnicka-Czerwiec J Bartosik-Psujek H. Spectral signature of multiple sclerosis. Preliminary studies of blood fraction by ATR FTIR technique. Biochem Biophys Res Commun. (2022) 593:40–5. doi: 10.1016/j.bbrc.2022.01.046, PMID: 35051781

[30] Alba-Arbalat S Andorra M Sanchez-Dalmau B Camos-Carreras A Dotti-Boada M Pulido-Valdeolivas I. In vivo molecular changes in the retina of patients with multiple sclerosis. Invest Ophthalmol Vis Sci. (2021) 62:11. doi: 10.1167/iovs.62.6.11, PMID: 33974046 PMC8114005

[31] Stoessel D Stellmann J-P Willing A Behrens B Rosenkranz SC Hodecker SC. Metabolomic profiles for primary progressive multiple sclerosis stratification and disease course monitoring. Front Hum Neurosci. (2018) 12:226. doi: 10.3389/fnhum.2018.00226, PMID: 29915533 PMC5994544

[32] Pieragostino D D’Alessandro M Di Ioia M Rossi C Zucchelli M Urbani A. An integrated metabolomics approach for the research of new cerebrospinal fluid biomarkers of multiple sclerosis. Mol BioSyst. (2015) 11:1563–72. doi: 10.1039/C4MB00700J, PMID: 25690641

[33] Fornasaro S Sergo V Bonifacio A. The key role of ergothioneine in label-free surface-enhanced Raman scattering spectra of biofluids: a retrospective re-assessment of the literature. FEBS Lett. (2022) 596:1348–55. doi: 10.1002/1873-3468.14312 35152417

[34] Al-Sammarraie SZ Bratchenko LA Typikova EN Lebedev PA Zakharov VP Bratchenko IA. Silver nanoparticles-based substrate for blood serum analysis under 785 nm laser excitation. J Biomed Photon Engineer. (2022) 8:010301. doi: 10.18287/JBPE22.08.010301

[35] Thompson AJ Banwell BL Barkhof F Carroll WM Coetzee T Comi G. Diagnosis of multiple sclerosis: 2017 revisions of the McDonald criteria. Lancet Neurol. (2018) 17:162–73. doi: 10.1016/S1474-4422(17)30470-2, PMID: 29275977

[36] Kurtzke JF. On the origin of EDSS. Mult Scler Relat Disord. (2015) 4:95–103. doi: 10.1016/j.msard.2015.02.003, PMID: 25787185

[37] Kurtzke JF. Clinical definition for multiple sclerosis treatment trials. Ann Neurol. (1994) 36:S73–9. doi: 10.1002/ana.410360717, PMID: 8017892

[38] Thompson AJ Montalban X Barkhof F Brochet B Filippi M Miller DH. Diagnostic criteria for primary progressive multiple sclerosis: a position paper. Ann Neurol. (2000) 47:831–5., PMID: 10852554

[39] Al-Sammarraie SZ Bratchenko LA Tupikova EN Skuratova MA Wang S Lebedev PA. Human blood plasma SERS analysis using silver nanoparticles for cardiovascular diseases detection. J Biomed Photon Engineer. (2024) 10:010301. doi: 10.18287/JBPE24.10.010301

[40] Khristoforova YA Bratchenko LA Skuratova MA Lebedeva EA Lebedev PA Bratchenko IA. Raman spectroscopy in chronic heart failure diagnosis based on human skin analysis. J Biophotonics. (2023) 16:e202300016. doi: 10.1002/jbio.202300016, PMID: 36999197

[41] Imitola J Côté D Rasmussen S Xie XS Liu Y Chitnis T. Multimodal coherent anti-stokes Raman scattering microscopy reveals microglia-associated myelin and axonal dysfunction in multiple sclerosis-like lesions in mice. J Biomed Opt. (2011) 16:021109. doi: 10.1117/1.3533312, PMID: 21361672 PMC3061329

[42] Poon KWC Brideau C Klaver R Schenk GJ Geurts JJ Stys PK. Lipid biochemical changes detected in normal appearing white matter of chronic multiple sclerosis by spectral coherent Raman imaging. Chem Sci. (2018) 9:1586–95. doi: 10.1039/C7SC03992A, PMID: 29675203 PMC5890326

[43] Tamosaityte S Galli R Uckermann O Sitoci-Ficici KH Koch M Later R. Inflammation-related alterations of lipids after spinal cord injury revealed by Raman spectroscopy. J Biomed Opt. (2016) 21:061008. doi: 10.1117/1.JBO.21.6.061008, PMID: 27146789

[44] Belogurov AA Kurkova IN Friboulet A Thomas D Misikov VK Zakharova MY. Recognition and degradation of myelin basic protein peptides by serum autoantibodies: novel biomarker for multiple sclerosis. J Immunol. (2008) 180:1258–67. doi: 10.4049/jimmunol.180.2.1258, PMID: 18178866

[45] Marro M Taubes A Abernathy A Balint S Moreno B Sanchez-Dalmau B. Dynamic molecular monitoring of retina inflammation by *in vivo* Raman spectroscopy coupled with multivariate analysis. J Biophotonics. (2014) 7:724–34. doi: 10.1002/jbio.201300101, PMID: 24019106

[46] Boerger M Funke S Leha A Roser A-E Wuestemann A-K Maass F. Proteomic analysis of tear fluid reveals disease-specific patterns in patients with Parkinson’s disease – a pilot study. Parkinsonism Relat Disord. (2019) 63:3–9. doi: 10.1016/j.parkreldis.2019.03.001, PMID: 30876839

[47] Kim A Nigmatullina R Zalyalova Z Soshnikova N Krasnov A Vorobyeva N. Upgraded methodology for the development of early diagnosis of Parkinson’s disease based on searching blood markers in patients and experimental models. Mol Neurobiol. (2019) 56:3437–50. doi: 10.1007/s12035-018-1315-2, PMID: 30128652

[48] Ralbovsky NM Lednev IK. Towards development of a novel universal medical diagnostic method: Raman spectroscopy and machine learning. Chem Soc Rev. (2020) 49:7428–53. doi: 10.1039/D0CS01019G, PMID: 32996518

[49] Khristoforova Y Bratchenko L Bratchenko I. Raman-based techniques in medical applications for diagnostic tasks: a review. Int J Mol Sci. (2023) 24:15605. doi: 10.3390/ijms242115605, PMID: 37958586 PMC10647591

[50] Karlov VA Savin AA Smertina LP Redchits EG Seleznev AN Svetaĭlo LI. Changes in rheological properties of blood in multiple sclerosis and their correction. Zh Nevropatol Psikhiatr Im S S Korsakova. (1990) 90:47–50. PMID: 1706550

[51] Francis PL Jakubovic R O’Connor P Zhang L Eilaghi A Lee L. Robust perfusion deficits in cognitively impaired patients with secondary-progressive multiple sclerosis. AJNR Am J Neuroradiol. (2013) 34:62–7. doi: 10.3174/ajnr.A3148, PMID: 22700746 PMC7966305

[52] Bratchenko LA Al-Sammarraie SZ Tupikova EN Konovalova DY Lebedev PA Zakharov VP. Analyzing the serum of hemodialysis patients with end-stage chronic kidney disease by means of the combination of SERS and machine learning. Biomed Opt Express. (2022) 13:4926–38. doi: 10.1364/BOE.455549, PMID: 36187246 PMC9484439

[53] Xiong C Zhong Q Yan D Zhang B Yao Y Qian W. Multi-branch attention Raman network and surface-enhanced Raman spectroscopy for the classification of neurological disorders. Biomed Opt Express. (2024) 15:3523–40. doi: 10.1364/BOE.514196, PMID: 38867772 PMC11166416

